# Prediction of various blood group systems using Korean whole-genome sequencing data

**DOI:** 10.1371/journal.pone.0269481

**Published:** 2022-06-03

**Authors:** Jungwon Hyun, Sujin Oh, Yun Ji Hong, Kyoung Un Park

**Affiliations:** 1 Department of Laboratory Medicine, Dongtan Sacred Heart Hospital, Hallym University College of Medicine, Hwaseong, South Korea; 2 Department of Laboratory Medicine, Seoul National University College of Medicine, Seoul, South Korea; 3 Department of Laboratory Medicine, Seoul National University Bundang Hospital, Seongnam, South Korea; Istanbul University-Cerrahpaşa, Cerrahpaşa Faculty of Medicine, TURKEY

## Abstract

**Aims:**

This study established blood group analysis methods using whole-genome sequencing (WGS) data and conducted blood group analyses to determine the domestic allele frequency using public data from the Korean whole sequence analysis of the Korean Reference Genome Project conducted by the Korea Disease Control and Prevention Agency (KDCA).

**Materials and methods:**

We analyzed the differences between the human reference sequences (hg19) and the conventional reference cDNA sequences of blood group genes using the Clustal Omega website, and established blood group analysis methods using WGS data for 41 genes, including 39 blood group genes involved in 36 blood group antigens, as well as the *GATA1* and *KLF1* genes, which are erythrocyte-specific transcription factor genes. Using CLC genomics Workbench 11.0 (Qiagen, Aarhus, Denmark), variant analysis was performed on these 41 genes in 250 Korean WGS data sets, and each blood group’s genotype was predicted. The frequencies for major alleles were also investigated and compared with data from the Korean rare blood program (KRBP) and the Erythrogene database (East Asian and all races).

**Results:**

Among the 41 blood group-related genes, hg19 showed variants in the following genes compared to the conventional reference cDNA: *GYPA*, *RHD*, *RHCE*, *FUT3*, *ACKR1*, *SLC14A1*, *ART4*, *CR1*, and *GCNT2*. Among 250 WGS data sets from the Korean Reference Genome Project, 70.6 variants were analyzed in 205 samples; 45 data samples were excluded due to having no variants. In particular, the *FUT3*, *GNCT2*, *B3GALNT1*, *CR1*, and *ACHE* genes contained numerous variants, with averages of 21.1, 13.9, 13.4, 9.6, and 7.0, respectively. Except for some blood groups, such as ABO and Lewis, for which it was difficult to predict the alleles using only WGS data, most alleles were successfully predicted in most blood groups. A comparison of allele frequencies showed no significant differences compared to the KRBP data, but there were differences compared to the Erythrogene data for the Lutheran, Kell, Duffy, Yt, Scianna, Landsteiner-Wiener, and Cromer blood group systems. Numerous minor blood group systems that were not available in the KRBP data were also included in this study.

**Conclusions:**

We successfully established and performed blood group analysis using Korean public WGS data. It is expected that blood group analysis using WGS data will be performed more frequently in the future and will contribute to domestic data on blood group allele frequency and eventually the supply of safe blood products.

## Introduction

Human red blood cells contain many blood group antigens. To date, 43 blood group systems containing 345 red cell antigens have been officially recognized by the International Society of Blood Transfusion (ISBT) [1]. The diversity of blood group antigens is primarily due to single nucleotide polymorphisms (SNP) in blood group genes. Blood group antigen typing is classically conducted using serological testing by hemagglutination, but recently this process has been automated. DNA-based molecular diagnostics (genotyping) have replaced serological methods. That is, SNP-based molecular testing and Sanger sequencing are used to analyze specific SNPs, alongside DNA microarray methods in clinical laboratories [2–4]. Molecular testing is easy to automate, can be multiplexed, and does not require expensive and difficult-to-find antisera, making it possible to test a broader range of blood types for patients and donors, and to help identify donors and select blood products for rare blood types [5]. However, SNP-based molecular diagnosis or Sanger sequencing have limitations as they cannot include all known blood group genes or detect new blood group antigen alleles. Several commercial multiplexed molecular diagnostic kits are currently available, but they do not cover all known blood group genes, and at most, they only identify 35–37 of red blood cell antigens from 10–11 blood groups. Erythrocyte genotyping using next-generation sequencing (NGS) has several advantages [6–8]. NGS enables the evaluation of whole-genome sequences to detect gene rearrangements and analyze copy numbers. NGS can detect new alleles in addition to known SNPs and establish new weak or silencing alleles. One study performed blood group analysis using NGS data from 2,504 people provided by the 1000 Genomes Project, but Korean data were not included [9].

The Korean rare blood program (KRBP), known as the Korean national recipient registry, was established in July 2013 [10, 11]. The definition of a rare blood group depends on the prevalence of blood antigens in a specific population. Accurate data on the frequencies of various blood antigens are essential for a rare blood program, which can then be used to predict the availability of blood products for use in patients with the corresponding antibodies. We used commercially available multiplex molecular assays to establish the rare donor program and explored the prevalence of various blood group antigens. However, not all known blood group antigens were included. The present study established blood group analysis methods using whole-genome sequencing (WGS) data and conducted blood group analyses to determine the domestic allele frequencies. These were compared with previous KRBP data and data from other ethnic groups using public data from the Korean whole-genome sequencing analysis of the Korean Reference Genome Project conducted by the Korea Disease Control and Prevention Agency (KDCA).

## Materials and methods

### Difference analysis between human reference sequences (hg19) and conventional reference cDNA

Conventional reference alleles and coding DNA sequences (CDS) were investigated for 41 genes (Table 1 and S1 Table), including 39 blood group genes involved in 36 blood group antigens, and the *GATA1* and *KLF1* genes, which are erythrocyte-specific transcription factor genes [12–14]. The conventional reference alleles for 40 genes were available directly from ISBT and *FUT3* alleles were available from the Blood Group Antigen Gene Mutation Database (dbRBC). The human reference genome (hg19) UCSC genomic transcripts (corresponding to the splicing pattern of the conventional cDNA sequence) for these 41 genes were also investigated using the UCSC genome browser [15]. The CDS of the conventional reference alleles and the human reference genomes for each gene were aligned, and the Clustal Omega website was used to identify nucleotide changes [16].We then analyzed the differences between hg19 and conventional reference cDNA and determined the blood group alleles of hg19 (Table 1). We described our overall work flow in S1 Fig.

**Table 1:** Prediction of various blood group systems of human reference genome (hg19) compared to conventional cDNA sequences analyzed using Clustal Omega.

ISBT No.	System name (symbol)	Gene name	Chromosomal location	Conventional reference allele	Conventional reference phenotype	Nucleotide change	Predicted amino acid change	Predicted allele name
001	ABO (ABO)	*ABO*	9q34.2	ABO*A1.*01*	A_1_	-	-	ABO*A1.*01*
002	MNS (MNS)	*GYPA*	4q31.21	GYPA*01	MNS:1 or M+	38C>A; 59C>T; 71G>A; 72T>G; 93C>T	Ala13Glu; Ser20Leu; Gly24Glu	*ND*
		*GYPB*		GYPB*04	MNS:4 or s+	-	-	GYPB*04
003	P1PK (P1PK)	*A4GALT*	22q13.2	A4GALT*01	P1+/–, Pk+	-	-	A4GALT*01
004	Rh (RH)	*RHD*	1p36.11	RHD*01	D RH:1	1136C>T	Thr379Met	RHD*10.00 RHD*DAU0
		*RHCE*		RHCE*01	RH:4 or c	48G>C	Trp16Cys	RHCE*01.*01*
					RH:5 or e			
					RH:6 or f (ce)			
005	Lutheran (LU)	*BCAM*	19q13.32	LU*02	LU:2 or Lu(b+)	-	-	LU*02
006	Kell (KEL)	*KEL*	7q34	KEL*02	KEL:2 or k+	-	-	KEL*02
007	Lewis (LE)	*FUT3*	19p13.3	*FUT3*	*ND*	202T>C; 314C>T	Trp68Arg; Thr105Met	*FUT3 202C*, *314T*
008	Duffy (FY)	*ACKR1*	1q23.2	FY*02	FY:2 or Fy(b+)	125A>G	Asp42Gly	FY*01
009	Kidd (JK)	*SLC14A1*	18q12.3	JK*02	JK:2 or Jk(b+)	838A>G	Asn280Asp	JK*01
010	Diego (DI)	*SLC4A1*	17q21.31	DI*02	DI:–1,2 or Di(a–b+)	-	-	DI*02
011	Yt (YT)	*ACHE*	7q22.1	YT*01	YT:1 or Yt(a+)	-	-	YT*01
012	Xg (XG)	*XG*	Xp22.33	XG*01	Xg(a+)	-	-	XG*01
013	Scianna (SC)	*ERMAP*	1p34.2	SC*01	SC:1 or Sc1+	-	-	SC*01
014	Dombrock (DO)	*ART4*	12p12.3	DO*01	DO:1+ or Do(a+)	378C>T; 624T>C; 793A>G	Asn265Asp	DO*02
015	Colton (CO)	*AQP1*	7p14.3	CO*01.*01*	CO:1 or Co(a+)	-	-	CO*01.*01*
016	Landsteiner-Wiener (LW)	*ICAM4*	19p13.2	LW*05	LW:5 or LW(a+)	-	-	LW*05
017	Chido/ Rodgers (CH/RG)	*C4A*	6p21.33	C4A*3	Ch-Rg+	-	-	C4A*3
					CH/RG:-1,-2,-3,-4,-5,-6,11,12			
		*C4B*		C4B*3	Ch+Rg-	-	-	C4B*3
					CH/RG:1,2,3,4,5,6,-11,-12			
018	H (H)	*FUT1*	19q13.33	FUT1*01	H+	-	-	FUT1*01
		*FUT2*		FUT2*01	H+	-	-	FUT2*01
019	Kx (KX)	*XK*	Xp21.1	XK*01	XK:1 or Kx+	-	-	XK*01
020	Gerbich (GE)	*GYPC*	2q14.3	GE*01	GE:2,3,4	-	-	GE*01
021	Cromer (CROM)	*CD55*	1q32.2	CROM*01	CROM:1 or Cr(a+)	-	-	CROM*01
022	Knops (KN)	*CR1*	1q32.2	KN*01	KN:1, KN:3, KN:4, KN:8, KN:9	180G>A; 4828T>A; 5905G>A	Ser1610Thr; Ala1969Thr	*ND*
023	Indian (IN)	*CD44*	11p13	IN*02	In(a–b+)	-	-	IN*02
024	Ok (OK)	*BSG*	19p13.3	OK*01.*01*	OK:1 or Ok(a+)	-	-	OK*01.*01*
025	Raph (RAPH)	*CD151*	11p15.5	RAPH*01	RAPH:1 or MER2+	-	-	RAPH*01
026	John Milton Hagen (JMH)	*SEMA7A*	15q24.1	JMH*01	JMH:1 or JMH+	-	-	JMH*01
027	I (I)	*GCNT2*	6p24.3-p24.2	GCNT2*01	I	816G>C	Glu272Asp	GCNT2*02
028	Globoside (GLOB)	*B3GALNT1*	3q26.1	GLOB*01	GLOB:1 (P+)	-	-	GLOB*01
029	Gill (GIL)	*AQP3*	9p13.3	GIL*01	GIL:1 or GIL+	-	-	GIL*01
030	Rh-associated glycoprotein (RHAG)	*RHAG*	6p12.3	RHAG*01	RHAG:1 or Duclos+	-	-	RHAG*01
031	FORS (FORS)	*GBGT1*	9q34.2	GBGT1*01N.*01*	FORS:–1 (FORS–)	-	-	GBGT1*01N.*01*
032	JR (JR)	*ABCG2*	4q22.1	ABCG2*01	Jr(a+)	-	-	ABCG2*01
033	LAN (LAN)	*ABCB6*	2q35	ABCB6*01	Lan+	-	-	ABCB6*01
034	Vel (VEL)	*SMIM1*	1p36.32	VEL*01	Vel+	-	-	VEL*01
035	CD59 (CD59)	*CD59*	11p13	CD59*01	CD59:+1 or CD59.1+	-	-	CD59*01
036	Augustine (AUG)	*SLC29A1*	6p21.1	AUG*01	At(a+) AUG:1	-	-	AUG*01
Associated genes		*GATA1*	Xp11.23	GATA1*01		-	-	GATA1*01
Associated genes		*KLF1*	19p13.13	KLF1*01		-	-	KLF1*01

ISBT, International Society of Blood Transfusion; ND, not determined

### Establishment of blood group analysis methods using WGS data

After importing the WGS data (BAM file) using CLC genomics Workbench 11.0 (Qiagen, Aarhus, Denmark) [17], the data were realigned to hg19, and variant analysis was performed on the coding regions of the 41 blood group-related genes. The alleles of each blood group were predicted by analyzing the variants for each gene and comparing them with the hg19 genotype.

### Blood group analysis using Korean WGS public data

We received the 250 Korean WGS data (BAM files) of the Korean Reference Genome Project through the Human Resource Distribution Desk of the National Institute of Health of the KDCA. Variant analysis was performed on 41 blood group-related genes in the Korean WGS data using the above method, and the alleles of each blood group were predicted. The frequencies of the major alleles were also investigated and compared with the frequencies in the previous KRBP data and the Erythrogene database (East Asians and all races) using data from 2,504 people from 26 races of the 1000 Genomes Project.

### Statistical analysis

Chi-square test and Fisher’s exact test were applied to compare the allele frequencies and data with *P* values <0.05 were considered statistically significant. Statistical analyses were performed using MedCalc software, version 19.8 (MedCalc Software Ltd., Ostend, Belgium)

### Ethics statement

This study uses public data, and since it uses already anonymized data, it does not contact the research subjects, uses information that has already been disclosed to the public and it was NGS data (BAM file) that did not include other medical records. This study was approved by the Institutional Ethics Committee of Seoul National University Bundang Hospital with waiver of consent and review exemption (IRB No. X-1801-447-906 and X-1903-528-901).

## Results

### Difference between hg19 and conventional reference cDNA

We investigated and analyzed the differences between the human reference sequences (hg19) and the CDS of the conventional reference alleles for 41 blood group-related genes. Table 1 lists the ISBT number, blood system name and symbol, gene name, chromosomal location, conventional reference allele, and phenotype for the 41 blood group-related genes analyzed in this study. Table 1 also lists the differences between hg19 and conventional cDNA for these 41 genes, including nucleotide changes, predicted amino acid changes, predicted allele name, and predicted phenotype. Among these 41 genes, hg19 showed variants in the following genes compared to the conventional reference cDNA: *GYPA*, *RHD*, *RHCE*, *FUT3*, *ACKR1*, *SLC14A1*, *ART4*, *CR1*, and *GCNT2*.

The conventional reference sequence for GYPA*01 encodes an M+ phenotype. The human reference genome *GYPA* sequence showsc.38C>A[p.Ala13Glu] and c.93C>T[p.Thr31Thr] variants in addition to the GYPA*02 allele encoding the N+ phenotype.

The *RHD* gene shows the c.1136C>T[p.Thr379Met] variant compared to the RHD*01 conventional reference allele, which corresponds to the RHD*DAU0 allele. RHCE*01, the conventional reference allele of the *RHCE* gene, shows a c+e+ phenotype. However, the human reference genome shows the c.48G>C[p.Trp16Cys] variant, which corresponds to the RHCE*01.*01* allele encoding a weak e phenotype.

Compared to the conventional reference allele, the *FUT3* gene shows the c.202T>C[p.Trp68Arg] and c.314C>T[p.Thr105Met] variants, and these variants encode the Le(a-b-) phenotype.

The *ACKR1* gene shows a variant in c.125A>G[p.Asp42Gly] compared to the FY*02 conventional reference allele encoding the Fy(b+) phenotype, corresponding to the FY*01 allele showing the Fy(a+) phenotype.

The *SCL14A1* gene shows a c.838A>G[p.Asn280 Asp] variant compared to the JK*02 conventional reference allele encoding the Jk(b+) phenotype, corresponding to the JK*01 allele showing the Jk(a+) phenotype.

Compared to the DO*01 conventional reference allele, which shows the Do(a+) phenotype, the *ART4* gene shows c.378C>T[p.Tyr126Tyr], c.624T>C[p.Leu208Leu], and c.793A>G[p.Asn265Asp] variants, which correspond to the DO*02 allele showing the Do(b+) phenotype.

Compared to the conventional reference allele, the *CR1* gene shows the c.180G>A, c.4828T>A[p.Ser1610Thr], and c.5905G>A[p.Ala1969Thr] variants. The c.4828T>A[p.Ser1610Thr] variant encodes a high-frequency Knops antigen (SI3) deficiency and exhibits a SI3-(SI:1,-2,-3) phenotype (KN:-8).

Compared to the conventional reference allele, the *GCNT2* gene shows a c.816G>C [p.Glu272Asp] variant, which corresponds to the GCNT2*02 allele and exhibits an I+ phenotype like GCNT2*01, the conventional reference allele.

### Blood group analysis using Korean WGS public data

Among the 250 WGS data sets of the Korean Reference Genome Project, an average of 70.6 variants were analyzed in 205 data samples. We excluded 45 data samples due to their showing no variants. The *FUT3*, *GNCT2*, *B3GALNT1*, *CR1*, and *ACHE* genes showed several variants, having an average of 21.1, 13.9, 13.4, 9.6, and 7.0 variants, respectively, making allele prediction for these genes difficult.

Other alleles were successfully predicted in most blood groups, except for ABO and Lewis, for which it was difficult to predict alleles and phenotypes from the WGS data alone. Table 2 lists the blood group system predictions from the representative data compared to conventional cDNA sequences.

**Table 2:** Prediction of various blood group systems in one representative data compared to conventional cDNA sequences analyzed using CLC Genomics Workbench.

ISBT No.	System name (symbol)	Gene name	Nucleotide change	Predicted amino acid change	Allele name
001	ABO (ABO)	*ABO*	646T>A; 681G>A; 771C>T; 829G>A	Phe216Ile; Val277Met	ABO*AW.*31*.*02–05*
002	MNS (MNS)	*GYPA*	-	-	*ND*
		*GYPB*	-	-	GYPB*04
003	P1PK (P1PK)	*A4GALT*	-	-	A4GALT*01
004	Rh (RH)	*RHD*	-	-	RHD*10.*00* RHD*DAU0
		*RHCE*	-	-	RHCE*01.*01*
005	Lutheran (LU)	*BCAM*	-	-	LU*02
006	Kell (KEL)	*KEL*	-	-	KEL*02
007	Lewis (LE)	*FUT3*	63A>G; 179G>A;189C>A; 201_202delACinsGT; 207A>G; 216_217delCCinsTA; 221_222delTCinsCA; 225T>C; 235T>C; 243T>C; 262A>G; 264A>G; 274C>A; 313_314delATinsGC; 344C>A; 351T>C; 353_355delAGTinsGTG; 366A>G; 407A>G; 409T>A; 415C>T; 417A>C; 421_423delCCTinsAGC; 431A>G; 451A>G; 732C>T; 1007A>C	Arg60His; Arg68Trp; His73Asn; Ile74Thr; Ser79Pro; Thr88Ala; His92Asn; Met105Ala; Ser115Tyr; Lys118_Ser119delinsSerAla; Asn136Ser; Leu137Met; Pro139Ser; Pro141Ser; Gln144Arg; Arg151Gly; Asp336Ala	*ND*
008	Duffy (FY)	*ACKR1*	-	-	FY*01
009	Kidd (JK)	*SLC14A1*	-	-	JK*01
010	Diego (DI)	*SLC4A1*	-	-	DI*02
011	Yt (YT)	*ACHE*	1790C>T; 1794G>C; 1796C>T; 1811A>T; 1814_1815delAGinsTC; 1818T>C; 1834_1835delTCinsCT; 1838A>T	Pro597Leu; Pro599Leu; His604Leu; Gln605Leu; His613Leu;	YT*01
012	Xg (XG)	*XG*	-	-	XG*01
013	Scianna (SC)	*ERMAP*	-	-	SC*01
014	Dombrock (DO)	*ART4*	-	-	DO*02
015	Colton (CO)	*AQP1*	-	-	CO*01.*01*
016	Landsteiner-Wiener (LW)	*ICAM4*	-	-	LW*05
017	Chido/Rodgers (CH/RG)	*C4A*	-	-	C4A*3
		*C4B*	-	-	C4B*3
018	H (H)	*FUT1*	-	-	FUT1*01
		*FUT2*	-	-	FUT2*01
019	Kx (KX)	*XK*	-	-	XK*01
020	Gerbich (GE)	*GYPC*	-	-	GE*01
021	Cromer (CROM)	*CD55*	-	-	CROM*01
022	Knops (KN)	*CR1*	747A>G; 1843_1844delCCinsAT; 3281G>A; 3321T>C; 3354T>A; 3357C>T; 3562C>A; 3568T>C; 4828A>T; 4843A>G	Pro615Ile; Arg1094His; Asn1118Lys; Pro1188Thr; Thr1610Ser; Ile1615Val	KN*01
023	Indian (IN)	*CD44*	-	-	IN*02
024	Ok (OK)	*BSG*	-	-	OK*01.*01*
025	Raph (RAPH)	*CD151*	-	-	RAPH*01
026	John Milton Hagen (JMH)	*SEMA7A*	-	-	JMH*01
027	I (I)	*GCNT2*	576A>G; 601C>A; 606G>A; 616C>T; 816C>G; 856C>T; 864A>G; 870A>C; 873A>G; 876T>C; 882T>C; 912T>C; 919G>A; 922T>C	His206Tyr; Asp272Glu; Val307Ile; Ser308Pro	GCNT*01
028	Globoside (GLOB)	*B3GALNT1*	131T>C; 136_138delCGCinsTAT; 141_142delGAinsAG; 156C>T; 159_160delTGinsCA; 391T>C; 434G>A; 526G>A; 528A>C; 589C>T; 675T>A; 838A>G; 890_891delTGinsCA; 904C>T; 906_907delGAinsTG; 910C>T	Ile44Thr; Arg46Tyr; Asn48Asp; Glu54Lys; Arg145Gln;Val176Ile; Asn280Asp; Leu297Ser; Arg303Gly; Arg304Cys	GLOB*01
029	Gill (GIL)	*AQP3*	-	-	GIL*01
030	Rh-associated glycoprotein (RHAG)	*RHAG*	-	-	RHAG*01
031	FORS (FORS)	*GBGT1*	728G>A	Arg243His	GBGT1*01N.*01*
032	JR (JR)	*ABCG2*	-	-	ABCG2*01
033	LAN (LAN)	*ABCB6*	117G>A	-	ABCB6*01
034	Vel (VEL)	*SMIM1*	-	-	VEL*01
035	CD59 (CD59)	*CD59*	-	-	CD59*01
036	Augustine (AUG)	*SLC29A1*	-	-	AUG*01
Associated genes		*GATA1*	-	-	GATA1*01
Associated genes		*KLF1*	304T>C	Ser102Pro	KLF1*BGM12

ISBT, International Society of Blood Transfusion; ND, not determined

**Fig 1:**
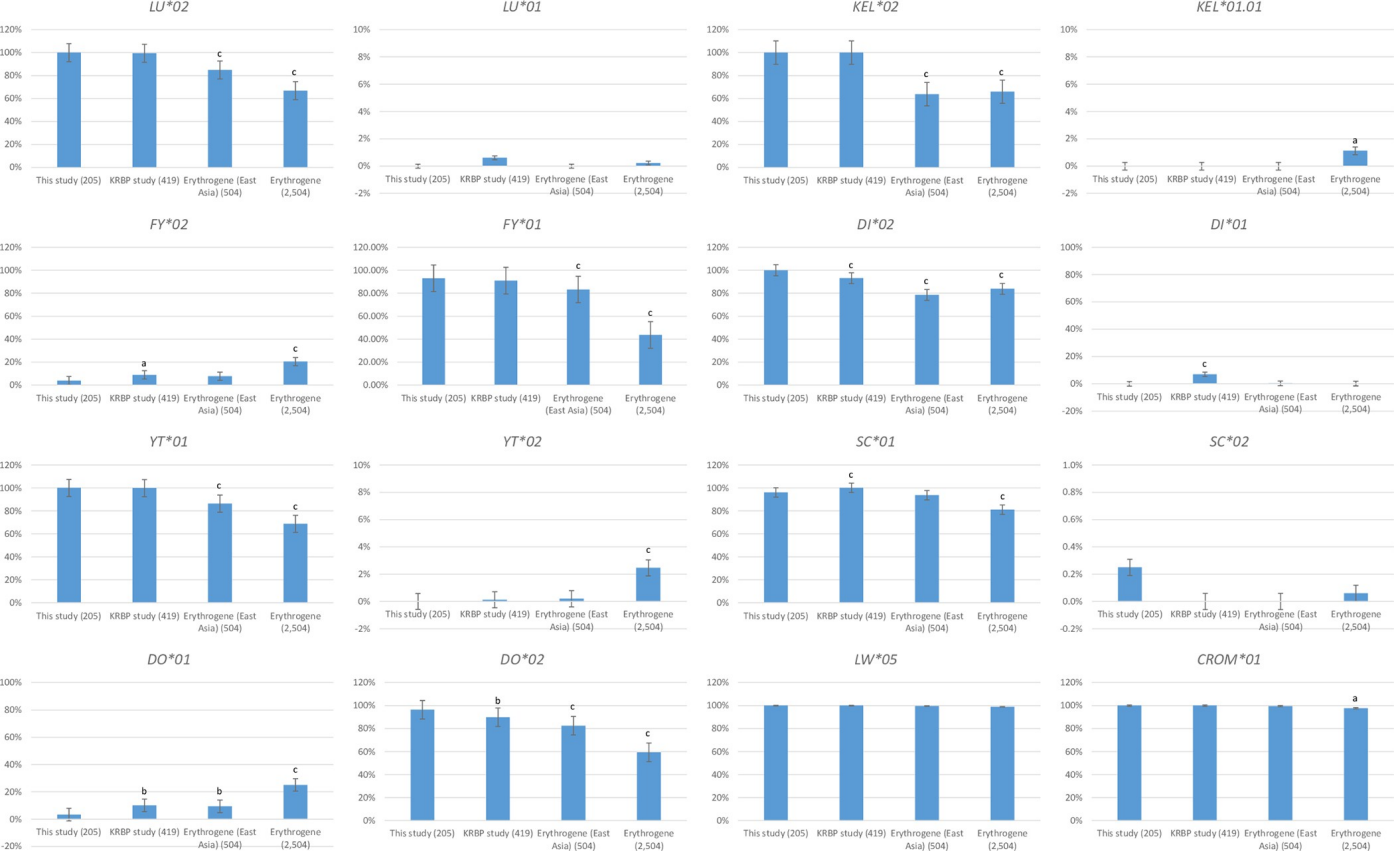
Comparison analysis of allele frequencies between this study, KRBP and Erythrogene data. Allele frequencies of Lutheran, Kell, Duffy (FY*01), Yt, Landsteiner-Wiener, and Cromer blood group systems showed no significant differences between this study and KRBP study. However, the allele frequencies of Lutheran, Kell, Duffy (FY*01), and Yt blood group systems showed significant differences from the Erythogene (East Asian and all races) data and allele frequency of Cromer blood group system showed significant difference from the Erythrogene (all races). The high-frequency alleles LU*02, KEL*02, FY*01, YT*01, SC*01, DO*02, and CROM*01 were more frequent in Koreans than in East Asians or all races in the Erythrogene database. ^a^P value <0.05 between this study compared to KRBP, Erythrogene (East Asia), or Erythrogene; ^b^P value <0.01 between this study compared to KRBP, Erythrogene (East Asia), or Erythrogene; ^c^P value <0.001 between this study compared to KRBP, Erythrogene (East Asia), or Erythrogene. Abbreviation: KRBP, Korean rare blood program.

Table 3 and Fig 1 show the allele frequencies compared with previous KRBP data sets and the Erythrogene (East Asian and all races) database. Allele frequencies were similar for the Lutheran, Kell, Duffy (FY*01), Yt, Landsteiner-Wiener, and Cromer blood group systems compared to the KRBP data, but there were differences compared to the Erythrogene data. This study included numerous minor blood group systems that were not available in the KRBP data, and most showed allele frequency differences compared to the Erythrogene data.

**Table 3:** Comparison of allele frequencies between this study, KRBP and Erythrogene data.

Blood group	Gene	Nucleotide changes	Predicted amino acid changes	Allele name	This study (205)	KRBP study (419)	Erythrogene (East Asia) (504)	Erythrogene (2,504)
Lutheran	*BCAM*	-	-	LU*02	100%	99.39%	84.82%^c^	66.77%^c^
		230G>A	Arg77His	LU*01	0%	0.61%	0%	0.22%
Kell	*KEL*	-	-	KEL*02	100%	100%	63.79%^c^	65.93%^c^
		578C>T	Thr193Met	KEL*01.*01*	0%	0%	0%	1.12%^a^
Duffy	*ACKR1*	-	-	FY*02	3.91%	8.95%^a^	7.74%	20.51%^c^
		125A>G	Asp42Gly	FY*01	93.02%	91.05%	83.23%^c^	43.65%^c^
		125A>G; 199C>T	Asp42Gly; Leu67Phe	*1000G only*	3.07%	*NA*	8.73%^b^	2.16%
Diego	*SLC4A1*	-	-	DI*02	100%	93.08%^c^	78.57%^c^	83.85%^c^
		2561C>T	Pro854Leu	DI*01	0%	6.92%^c^	0.30%	0.18%
Yt	*ACHE*	-	-	YT*01	100%	99.88%	86.31%^c^	68.77%^c^
		1057C>A	His353Asn	YT*02	0%	0.12%	0.20%	2.46%^c^
Xg	*XG*	-	-	XG*01	100%	*NA*	99.87%	95.60%^b^
Scianna	*ERMAP*	-	-	SC*01	96.09%	100%^c^	93.75%	81.07%^c^
		169G>A	Gly57Arg	SC*02	0.28%	0%	0%	0.06%
		835G>A; 1248C>T	Val279Ile	*1000G only*	0.28%	*NA*	1.19%	0.26%
		976A>G	Asn326Asp	*1000G only*	3.35%	*NA*	1.79%	0.66%^c^
Dombrock	*ART4*	-	-	DO*01	3.35%	10.14%^b^	9.42%^b^	25.10%^c^
		793A>G	Asn265Asp	DO*02	96.37%	89.86%^b^	82.54%^c^	59.37%^c^
Colton	AQP1	-	-	CO*01.*01*	100%	*NA*	99.01%	95.61%^b^
		134C>T	Ala45Val	CO*02	0%	*NA*	0%	1.14%^a^
Landsteiner-Wiener	*ICAM4*	-	-	LW*05	100%	100%	99.70%	98.96%
Chido/Rogers	*C4A*	-	-	C4A*3	100%	*NA*	30.26%^c^	30.93%^c^
	*C4B*	-	-	C4B*3	100%	*NA*	99.40%	99.50%
H	*FUT1*	-	-	FUT1*01	91.62%	*NA*	67.66%^c^	89.22%^c^
		35C>T	Ala12Val	FUT1*02	7.82%	*NA*	31.25%^c^	9.82%
Kx	*XK*	-	-	XK*01	100%	*NA*	100%	99.92%
Gerbich	*GYPC*	-	-	GE*01	100%	*NA*	99.50%	97.40%^a^
Cromer	*CD55*	-	-	CROM*01	100%	100%	99.60%	97.72%^a^
Indian	*CD44*	-	-	IN*02	100%	*NA*	99.80%	99.04%
Ok	*BSG*	-	-	OK*01.*01*	100%	*NA*	97.62%^a^	98.20%
Raph	*CD151*	-	-	RAPH*01	100%	*NA*	98.91%	98.94%
John Milton Hagen	*SEMA7A*	-	-	JMH*01	100%	*NA*	99.11%	96.37%^b^
FORS	*GBGT1*	-	-	GBGT1*01N.*01*	94.14%	*NA*	45.44%^c^	59.96%^c^
		397G>A	Glu133Lys	*1000G only*	1.95%	*NA*	0.20%^c^	0.04%^c^
		707G>A	Arg236His	*1000G only*	2.93%	*NA*	0.50%^c^	0.12%^c^
		728G>A	Arg243His	*1000G only*	0.98%	*NA*	0.30%	0.10%^b^
JR	*ABCG2*	34G>A	Val12Met	*1000G only*	2.44%	*NA*	29.66%^c^	13.68%^c^
		167G>A	Arg56Gln	*1000G only*	0.49%	*NA*	0%	0.02%^a^
Vel	SMIM1	-	-	VEL*01	100%	*NA*	*99*.*90%*	99.96%
Associated genes	*KLF1*	-	-	KLF*01	73.66%	*NA*	30.75%^c^	53.53%^c^
		304T>C	Ser102Pro	KF1*BGM12	22.93%	*NA*	59.23%^c^	37.88%^c^
		325C>T	Pro109Ser	*1000G only*	1.95%	*NA*	7.64%^c^	1.54%
		304T>C; 544T>C	Ser102Pro; Phe182Leu	*1000G only*	0.98%	*NA*	1.69%	4.89%^c^

KRBP, Korean rare blood program; NA, not available

^a^*P* value <0.05 between this study compared to KRBP, Erythrogene (East Asia), or Erythrogene

^b^*P* value <0.01 between this study compared to KRBP, Erythrogene (East Asia), or Erythrogene

^c^*P* value <0.001 between this study compared to KRBP, Erythrogene (East Asia), or Erythrogene

## Discussion

This study established blood group analysis methods using WGS data and analyzed the blood groups using Korean WGS public data. Blood group gene analysis differs from the genetic analysis used to diagnose tumors or congenital genetic diseases. There is a conventional reference allele for each blood group, and the SNPs and blood group genotypes according to the reference allele are well documented. There are several blood group gene databases. The Blood Group Antigen Mutation (BGMUT) Database was created by the Human Genome Variation Society (HGVS) in 1999 [14]. Since 2006, it has been operated by the National Institutes of Health (NIH) as part of the database Red Blood Cells (dbRBC) of the National Center for Biotechnology Information (NCBI), which ceased operation in October 2017. In 2016, Moller et al. created the Erythrogene database following analysis of 36 blood groups from the 1000 Genomes Project [9]. There are also the ISBT website [13], the Blood Group Antigen FactsBook [18], and BOOGIE [19]. Since the blood group genotypes of hg19 are not the same as the conventional reference alleles of each blood group, we first analyzed the blood group genotypes of the human reference genome and noted the differences compared to the conventional reference alleles. Since most variant analysis software is designed to find variants by comparing the nucleotide sequences to the human reference genome, variant analysis is performed on blood group-related genes in the same way as other genetic analyses. Therefore, the results of variant detection alone cannot determine the blood group types. In this study, the differences between hg19 and conventional cDNA were analyzed first (Table 1), and we used these results to conduct blood group analyses in the WGS data. WGS data analysis, similar to other NGS data analyses, undergoes the same process of read mapping and variant detection after aligning the sequences to the human reference sequence. Then the alleles are analyzed alongside the phenotype of each blood group using the detected variants.

Alleles were successfully predicted in most blood groups except for ABO (*ABO*) and Lewis (*FUT3*), which were difficult to predict using WGS data alone. ABO and Lewis antigens are carbohydrate antigens synthesized by enzymes [20]. The A and B genes of the ABO blood group are alleles located in the same position; having a blood type A means having the phenotype of A, but even with the same phenotype, the genotype may be *AA* or *AO*. Over 200 genotypes have been reported for *ABO*, which include nucleotide changes in various regions. However, the vcf file obtained following variant detection cannot distinguish the two alleles. Moreover, because carbohydrate antigen prediction requires the evaluation of several related genes, and the alleles associated with carbohydrate antigens are complex [5], determination of alleles based only on WGS data is challenging, and support is needed to establish the phenotyping results. It was difficult to determine the blood type using only the WGS data for the Lewis blood group, for which factors other than *FUT2* and *FUT3* genes are involved in the blood group phenotype.

Additionally, none of the variants of the RH (*RHD* and *RHCE*) and MNS (*GYPA* and *GYPB*) blood system were detected in the Korean public WGS data. However, we visually confirmed that the mapping and variant detection of these genes were performed successfully. Although the data were not included in this study, we also conducted the blood group analysis using the WGS data from clinical samples, and more variants were detected. The public data used was obtained from the Human Resource Distribution Desk of the National Institute of Health of the KDCA. These data sets were collected through the Korean Reference Genome Project between 2012 and 2014 using HiSeq2000 (Illumina, San Diego, CA, USA) analysis with a maximum of 30× depth coverage per sample [21]. The BAM files were already aligned to hg19 and we used hg19 as the reference genome. We judged that insufficient variant data was detected due to the lower coverage and poorer data quality compared to the clinical patient samples. High numbers of variants were detected in the *ACHE* (Yt), *CR1* (Knops), *GCNT2* (I), and *B3GALNT1*(Globoside) genes. However, it is not known whether most of these nucleotide changes encode antigenic epitopes [13], and we were able to predict the alleles in most cases (Table 2).

We also investigated the frequencies of the major alleles and compared them to the frequencies of the KRBP and Erythrogene (East Asian and all races) data from the 1000 Genomes Project (Table 3). The 1000 Genomes Project contains data from 2,504 people from 26 races, divided into African (661), American (347), East Asian (504), European (503), and South Asian (489); however, the East Asians only include Chinese, Japanese, and Vietnamese people, but not Koreans [9]. Although it is well known that allele frequencies vary among ethnic groups, few studies exist on the allele frequencies in different blood group systems [10, 22]. Allele frequencies of Lutheran, Kell, Duffy, Diego, Yt, Scianna, Dombrock, Landsteiner-Wiener, and Cromer blood group systems were available in the KRBP data and Lutheran, Kell, Duffy (FY*01), Yt, Landsteiner-Wiener, and Cromer blood group systems showed no significant differences between this study and KRBP study in allele frequencies. However, the allele frequencies of Lutheran, Kell, Duffy (FY*01), and Yt blood group systems showed significant differences from the Erythogene (East Asian and all races) data and allele frequency of Cromer blood group system showed significant difference from the Erythrogene (all races). The high-frequency alleles LU*02, KEL*02, FY*01, YT*01, SC*01, DO*02, and CROM*01 were more frequent in Koreans than in East Asians or all races in the Erythrogene database. In the Duffy system, the allele frequency of FY*02 showed significant difference from the KRBP data (*P* = 0.023), but this was because the allele with 125A>G and 199C>T nucleotide changes was detected as the FY*02 allele in the KRBP study. In the Diego system, the prevalence of the Di^a^ antigen is extremely rare in people of European or African descent, but is about 5% in people of Chinese or Japanese ancestry and has an even higher prevalence in the indigenous peoples of North and South America, reaching 54% [20]. The antigen prevalence of Di^a^ is 6.4–14.5% in Koreans [23]. The allele frequency of DI*01 was 6.92% in the KRBP data, 0.30% in the East Asian Erythrogene data, and 0.18% in all the Erythrogene data. However, no DI*01 variants were detected in the Korean public WGS data. This could be due to the smaller sample size or poor quality of the WGS data. In the Dombrock system, the frequency of the DO*01 allele is lower in this study than in the KRBP study because the hg19 itself is DO*02, so the variant may not have been detected due to the low data quality. Numerous minor blood group systems that were not included in the KRBP data are included in our study. Antibodies to many of these antigens are rarely encountered because they are high-prevalence antigens in most populations; however, the Colton, Gerbich, RHAG, JR, LAN, and Vel systems can cause acute hemolytic transfusion reactions or hemolytic diseases in newborns [24, 25]. Accurate data on the frequencies of various blood group antigens are essential to predict the availability of compatible blood components for use in patients with the corresponding antibodies and are indispensable for rare blood program. Accurate information on the frequencies of specific antigen-negative blood units will help reduce unnecessary antigen testing and avoid delays in issuing blood units to patients. Furthermore, it will contribute to improving blood transfusion safety and better blood supply management.

## Conclusion

We successfully established blood group analysis methods using WGS data and performed blood group analyses on Korean public WGS data. There were some limitations in this study in terms of the number and quality of the WGS data sets. Also, additional tests such as serologic tests or further molecular assays could not be performed. Nevertheless, even using WGS or whole-exome sequencing (WES) data, which is not intended for blood group genotyping, we were able to analyze the various blood group alleles using the method established in this study. In addition, accumulating frequency data for diverse blood group systems will enable safe blood products and the provision of adequate blood supplies for patients with the relevant antibodies.

## Supporting information

S1 Table The source of the conventional reference alleles for the 41 blood group genes. (DOCX)

S1 Fig Graphic work flow of this study. Part 1: Conventional reference alleles and coding DNA sequences (CDS) were investigated for 41 genes, including 39 blood group genes involved in 36 blood group antigens, and the *GATA1* and *KLF1* genes, which are erythrocyte-specific transcription factor genes(12). The human reference genome (hg19) UCSC genomic transcripts (corresponding to the splicing pattern of the conventional CDS) for these 41 genes were also investigated using the UCSC genome browser(15). The CDS of the conventional reference alleles and the human reference genomes for each gene were aligned, and the Clustal Omega website was used to identify nucleotide changes(16).We then analyzed the differences between hg19 and conventional reference cDNA and determined the blood group alleles of hg19. Part 2: After importing the 250 Korean WGS data (BAM) using CLC genomic Workbench 11.0 (Qiagen, Aarhus, Denmark)(17), the data were realigned to hg19, and variant analysis was performed on the coding regions of the 41 blood group-related genes. The alleles of each blood group were predicted by analyzing the variants for each gene and comparing them with the hg19 genotype. Abbreviations: CDS, coding DNA sequences; WGS, whole-genome sequencing.
(TIF)

S1 File The variant analysis file of 250 data. (XLSX)
