# Supplementary material for: Prediction of various blood group systems using Korean whole-genome sequencing data
Source: PLoS One. 2022 Jun 3;17(6):e0269481. doi: 10.1371/journal.pone.0269481 (PMC9165885; doi:10.1371/journal.pone.0269481)
Supplement: S1 Table — (DOCX) [file pone.0269481.s001.docx]

**S1 Table. The source of the conventional reference alleles for the 41 blood group genes.**

| ISBT No. | System name (symbol) | Gene name | Chromosomal location | Conventional reference allele | Source of reference alleles |
| --- | --- | --- | --- | --- | --- |
| 001 | ABO (ABO) | *ABO* | 9q34.2 | *ABO*A1.01* | ISBT v1.1 171023 |
| 002 | MNS (MNS) | *GYPA* | 4q31.21 | *GYPA*01* | ISBT v4.1 170119 |
|  |  | *GYPB* |  | *GYPB*04* |  |
| 003 | P1PK (P1PK) | *A4GALT* | 22q13.2 | *A4GALT*01* | ISBT v3.1 170105 |
| 004 | Rh (RH) | *RHD* | 1p36.11 | *RHD*01* | ISBT v6.0 211130 |
|  |  | *RHCE* |  | *RHCE*01* | ISBT v6.1 210823 |
| 005 | Lutheran (LU) | *BCAM* | 19q13.32 | *LU*02* | ISBT v5.0 200301 |
| 006 | Kell (KEL) | *KEL* | 7q34 | *KEL*02* | ISBT v7.0 211130 |
| 007 | Lewis (LE) | *FUT3* | 19p13.3 | *FUT3* | dbRBC 2017-05-24 |
| 008 | Duffy (FY) | *ACKR1* | 1q23.2 | *FY*02* | ISBT v6.1 211130 |
| 009 | Kidd (JK) | *SLC14A1* | 18q12.3 | *JK*02* | ISBT v7.0 210630 |
| 010 | Diego (DI) | *SLC4A1* | 17q21.31 | *DI*02* | ISBT v4.0 210630 |
| 011 | Yt (YT) | *ACHE* | 7q22.1 | *YT*01* | ISBT v7.0 200301 |
| 012 | Xg (XG) | *XG* | Xp22.33 | *XG*01* | ISBT v3.0 201030 |
| 013 | Scianna (SC) | *ERMAP* | 1p34.2 | *SC*01* | ISBT v4.0 210630 |
| 014 | Dombrock (DO) | *ART4* | 12p12.3 | *DO*01* | ISBT v6.0 211130 |
| 015 | Colton (CO) | *AQP1* | 7p14.3 | *CO*01.01* | ISBT v3.0 190418 |
| 016 | Landsteiner-Wiener (LW) | *ICAM4* | 19p13.2 | *LW*05* | ISBT v3.0 160623 |
| 017 | Chido/Rodgers (CH/RG) | *C4A* | 6p21.33 | *C4A*3* | ISBT v1.0 210630 |
|  |  | *C4B* |  | *C4B*3* |  |
| 018 | H (H) | *FUT1* | 19q13.33 | *FUT1*01* | ISBT v5.2 190418 |
|  |  | *FUT2* |  | *FUT2*01* |  |
| 019 | Kx (KX) | *XK* | Xp21.1 | *XK*01* | ISBT v5.0 210630 |
| 020 | Gerbich (GE) | *GYPC* | 2q14.3 | *GE*01* | ISBT v4.1 211130 |
| 021 | Cromer (CROM) | *CD55* | 1q32.2 | *CROM*01* | ISBT v3.0 160622 |
| 022 | Knops (KN) | *CR1* | 1q32.2 | *KN*01* | ISBT v3.0 160704 |
| 023 | Indian (IN) | *CD44* | 11p13 | *IN*02* | ISBT v6.1 190822 |
| 024 | Ok (OK) | *BSG* | 19p13.3 | *OK*01.01* | ISBT v4.0 190410 |
| 025 | Raph (RAPH) | *CD151* | 11p15.5 | *RAPH*01* | ISBT v5.0 201030 |
| 026 | John Milton Hagen (JMH) | *SEMA7A* | 15q24.1 | *JMH*01* | ISBT v5.0 211130 |
| 027 | I (I) | *GCNT2* | 6p24.3-p24.2 | *GCNT2*01* | ISBT v4.0 190408 |
| 028 | Globoside (GLOB) | *B3GALNT1* | 3q26.1 | *GLOB*01* | ISBT v4.0 190408 |
| 029 | Gill (GIL) | *AQP3* | 9p13.3 | *GIL*01* | ISBT v4.1 211130 |
| 030 | Rh-associated glycoprotein (RHAG) | *RHAG* | 6p12.3 | *RHAG*01* | ISBT v6.2 211130 |
| 031 | FORS (FORS) | *GBGT1* | 9q34.2 | *GBGT1*01N.01* | ISBT v1.0 170822 |
| 032 | JR (JR) | *ABCG2* | 4q22.1 | *ABCG2*01* | ISBT v4.0 190408 |
| 033 | LAN (LAN) | *ABCB6* | 2q35 | *ABCB6*01* | ISBT v4.0 170828 |
| 034 | Vel (VEL) | *SMIM1* | 1p36.32 | *VEL*01* | ISBT v2.1 211130 |
| 035 | CD59 (CD59) | *CD59* | 11p13 | *CD59*01* | ISBT v3.0 211130 |
| 036 | Augustine (AUG) | *SLC29A1* | 6p21.1 | *AUG*01* | ISBT v1.0 190408 |
| Associated genes | | *GATA1* | Xp11.23 | *GATA1*01* | ISBT v1.0 210630 |
| Associated genes | | *KLF1* | 19p13.13 | *KLF1*01* | ISBT v1.0 210630 |
