# Supplementary figures and images for: Prediction of various blood group systems using Korean whole-genome sequencing data
Source: PLoS One. 2022 Jun 3;17(6):e0269481. doi: 10.1371/journal.pone.0269481 (PMC9165885; doi:10.1371/journal.pone.0269481)

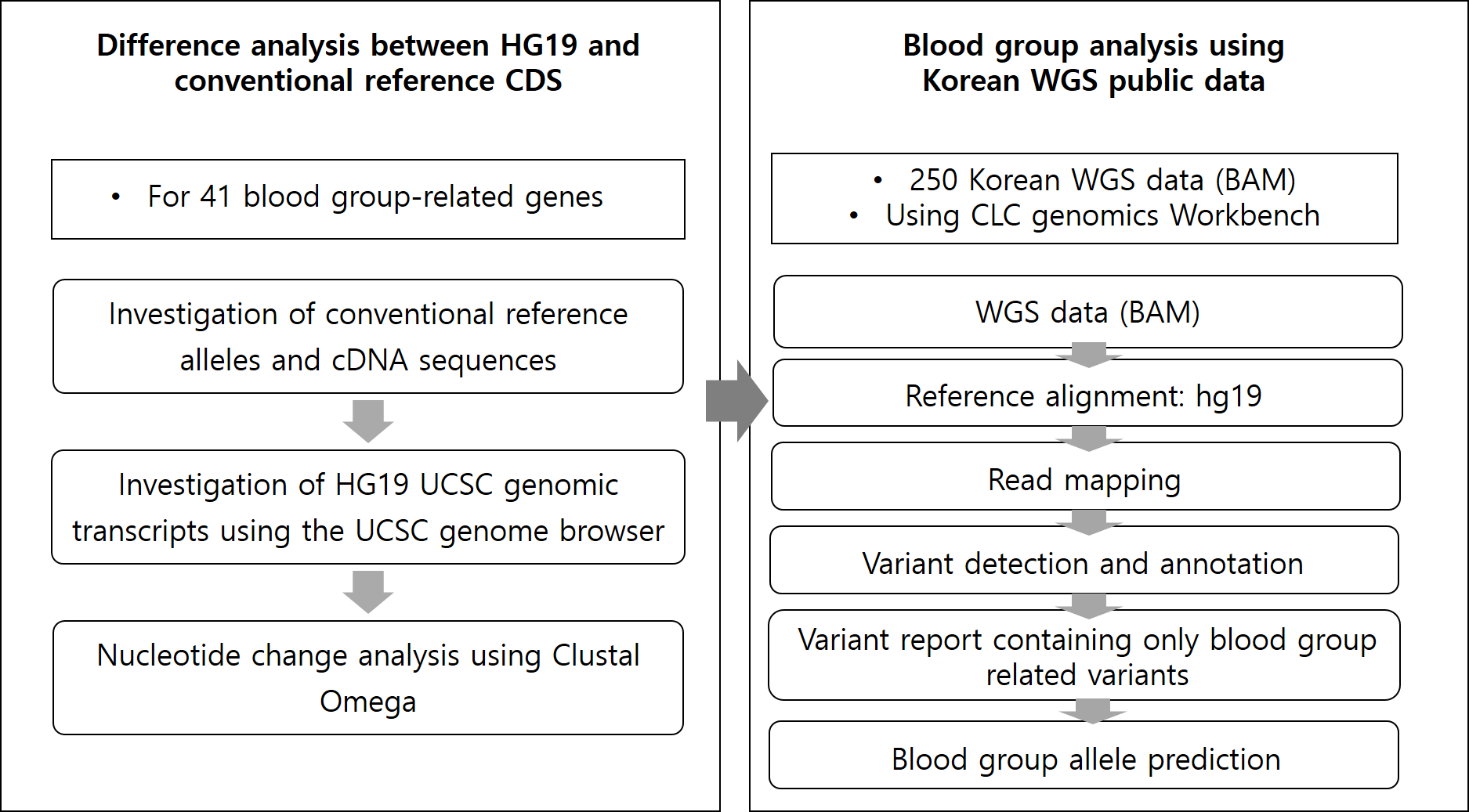

Supplement: S1 Fig — Part 1: Conventional reference alleles and coding DNA sequences (CDS) were investigated for 41 genes, including 39 blood group genes involved in 36 blood group antigens, and the GATA1 and KLF1 genes, which are erythrocyte-specific transcription factor genes(12). The human reference genome (hg19) UCSC genomic transcripts (corresponding to the splicing pattern of the conventional CDS) for these 41 genes were also investigated using the UCSC genome browser(15). The CDS of the conventional reference alleles and the human reference genomes for each gene were aligned, and the Clustal Omega website was used to identify nucleotide changes(16).We then analyzed the differences between hg19 and conventional reference cDNA and determined the blood group alleles of hg19. Part 2: After importing the 250 Korean WGS data (BAM) using CLC genomic Workbench 11.0 (Qiagen, Aarhus, Denmark)(17), the data were realigned to hg19, and variant analysis was performed on the coding regions of the 41 blood group-related genes. The alleles of each blood group were predicted by analyzing the variants for each gene and comparing them with the hg19 genotype. Abbreviations: CDS, coding DNA sequences; WGS, whole-genome sequencing. (TIF) [file pone.0269481.s002.tif]
